# 110 Years of *Avipoxvirus* in the Galapagos Islands

**DOI:** 10.1371/journal.pone.0015989

**Published:** 2011-01-13

**Authors:** Patricia G. Parker, Elizabeth L. Buckles, Heather Farrington, Kenneth Petren, Noah K. Whiteman, Robert E. Ricklefs, Jennifer L. Bollmer, Gustavo Jiménez-Uzcátegui

**Affiliations:** 1 Department of Biology, University of Missouri – St. Louis, St. Louis, Missouri, United States of America; 2 WildCare Institute, Saint Louis Zoo, St. Louis, Missouri, United States of America; 3 Department of Biomedical Sciences, College of Veterinary Medicine, Cornell University, Ithaca, New York, United States of America; 4 Department of Biological Sciences, University of Cincinnati, Cincinnati, Ohio, United States of America; 5 Department of Vertebrates, Charles Darwin Foundation, Puerto Ayora, Ecuador; Centers for Disease Control and Prevention, United States of America

## Abstract

The role of disease in regulating populations is controversial, partly owing to the absence of good disease records in historic wildlife populations. We examined birds collected in the Galapagos Islands between 1891 and 1906 that are currently held at the California Academy of Sciences and the Zoologisches Staatssammlung Muenchen, including 3973 specimens representing species from two well-studied families of endemic passerine birds: finches and mockingbirds. Beginning with samples collected in 1899, we observed cutaneous lesions consistent with *Avipoxvirus* on 226 (6.3%) specimens. Histopathology and viral genotyping of 59 candidate tissue samples from six islands showed that 21 (35.6%) were positive for *Avipoxvirus*, while alternative diagnoses for some of those testing negative by both methods were feather follicle cysts, non-specific dermatitis, or post mortem fungal colonization. Positive specimens were significantly nonrandomly distributed among islands both for mockingbirds (San Cristobal vs. Espanola, Santa Fe and Santa Cruz) and for finches (San Cristobal and Isabela vs. Santa Cruz and Floreana), and overall highly significantly distributed toward islands that were inhabited by humans (San Cristobal, Isabela, Floreana) vs. uninhabited at the time of collection (Santa Cruz, Santa Fe, Espanola), with only one positive individual on an uninhabited island. Eleven of the positive specimens sequenced successfully were identical at four diagnostic sites to the two canarypox variants previously described in contemporary Galapagos passerines. We conclude that this virus was introduced late in 1890′s and was dispersed among islands by a variety of mechanisms, including regular human movements among colonized islands. At present, this disease represents an ongoing threat to the birds on the Galapagos Islands.

## Introduction

Extinction risk associated with disease remains largely hypothetical [1] despite the attention that this problem receives [2]. Undisputed examples come from the Hawaiian Islands, where extinctions of endemic forest birds are attributed to avian pox and avian malaria transmitted from introduced species [1], [3].

*Avipoxvirus* is a pathogen of extreme concern in insular populations of birds [4]–[6]. The disease ‘pox’ is caused by a DNA virus (genus *Avipoxvirus*: Poxviridae). Its recognized strains vary in virulence and host specificity; the best-studied strains are those infecting passerine birds (canarypox virus) and galliform birds (fowlpox virus). The most common lesions associated with infection are epidermal nodules on feet, legs, and tissue surrounding the bill and eyes that may become ulcerated and enlarged to impede sight, feeding, and mobility. The less common diphtheritic form produces lesions inside respiratory and digestive systems, inhibiting breathing and swallowing [7]. Infective virions persist in the environment and enter through breaks in the skin, and are mechanically vectored by biting insects. Individuals that survive an aggressive infection often present deformed or missing digits, feet, or bills.

Current understanding of the extinctions of Hawaiian endemic birds involves a complex interaction of *Avipoxvirus*, a *Plasmodium* blood parasite, and their arthropod vectors [8]. Even in this case, the evidence for the role played by the pathogens in population declines and extinctions is largely indirect and circumstantial, derived from the introductions of *Avipoxvirus* and *Culex quinquefasciatus* mosquitoes in the 1800′s, followed by a sharp decline in bird numbers, and the subsequent introduction of the malaria agent *Plasmodium relictum*, causing yet further drastic declines and extinctions [5], [8]–[10]. The observation that mosquito-free higher elevations were a refuge for birds was crucial to understanding the transmission dynamics of these pathogens [10]. A specimen from 1900 was recently confirmed to have been infected with one of the virus strains known to be present in Hawaii today, but the presence of other canarypox virus strains suggests that there had been multiple introductions of at least two forms of the virus infecting the passerine birds, both distinct from the fowlpox virus infecting domestic fowl [11] in Hawaii. In contrast, two very similar strains have been described in passerine birds from the Galapagos Islands, both within the canarypox virus cluster, and similarly distinct from the fowlpox virus found in Galapagos chickens [12], suggesting the possibility of a single introduction of the canarypox virus, separate from introduction(s) of fowlpox virus.

The Galapagos Islands straddle the equator 1000 km west of Ecuador and comprise 19 major islands; 97 percent of the archipelago has been protected as a National Park and UNESCO World Heritage Site since 1959, and the archipelago retains almost its entire fauna [13]. However, pox-like symptoms occur in Galapagos endemic birds, including mockingbirds (*Mimus* spp.), doves (*Zenaida galapagoensis*), yellow warblers (*Dendroica petechia*), and some finches (*Geospiza* and *Camarhynchus* spp.). During the 1982–1983 El Niño Southern Oscillation (ENSO) event, Galapagos mockingbirds displaying pox-like lesions suffered significantly higher mortality than asymptomatic birds on Genovesa [14] and Santa Cruz [15]. Since then, pox-like symptoms have been reported in endemic birds from most major islands [12], [14], [16], [17]. It is unknown how long the canarypox virus variants infecting extant populations of birds have been in Galapagos.

The California Academy of Sciences (CAS) holds 1170 Galapagos avian specimens collected by R.E. Snodgrass and E. Heller in 1898 and 1899, and 7401 collected by R.H. Beck, E.W. Gifford and J.S. Hunter on the second Webster-Harris expedition in 1905 and 1906. Across both expeditions, 5580 specimens represent two passerine taxa in the archipelago: the finches (at least 13 endemic species), and the mockingbirds (four endemic species), from all 19 major islands. In these same taxa, the Zoologisches Staatssammlung Muenchen (ZSM) holds 130 specimens collected during an 1891 expedition by G. Bauer (by way of the Rothschild collection at Tring, United Kingdom), and another 161 specimens collected in 1897 on the first Webster-Harris Expedition (also by way of the Rothschild collection).

Only Floreana (colonized in 1807) was inhabited when Darwin visited in 1835, but the human population on Floreana was intermittent until the 1930′s, since which time it has been inhabited continuously. Today, five islands are inhabited. Floreana, San Cristobal (inhabited continuously since 1837), Isabela (inhabited continuously since 1893), Santa Cruz and its satellite island Baltra (Santa Cruz inhabited continuously since 1920) [18] are now home to more than 20,000 people. Throughout this time and prior to human inhabitation, many of the islands were visited by whalers, buccaneers, hunters, and more recently by scientific researchers. While there have been no known extinctions of bird species on the Galapagos Islands, the population (island) level extinction rate is approximately 100 times higher since human colonization than before, estimated from analysis of subfossil remains [19]–[21]. It is important to understand the history of *Avipoxvirus* in wild bird populations on Galapagos to assess its contribution to this accelerated population-level extinction rate. Because of the recent arrival and controlled spread of humans on the archipelago, we were able to use this extensive museum collection to ask whether the arrival and distribution of *Avipoxvirus* on the Galapagos Islands was associated with their inhabitation by humans.

## Methods

### Museum Collection

We (PGP, JLB, GJU) visited the CAS in June 2004 and again in June 2008 and examined 4313 of the 6371 passerine specimens collected between 1898 and 1906, including 2903 finches and 704 mockingbirds for cutaneous nodules consistent with poxvirus. Wearing gloves to prevent cross contamination of specimens, we inspected all skin on legs, feet, and around bills, and lightly ruffled feathers to expose other nodules. RER visited the ZSM in 2007 and similarly inspected the 266 finch and mockingbird specimens collected between 1891 and 1897.

**Figure 1:**
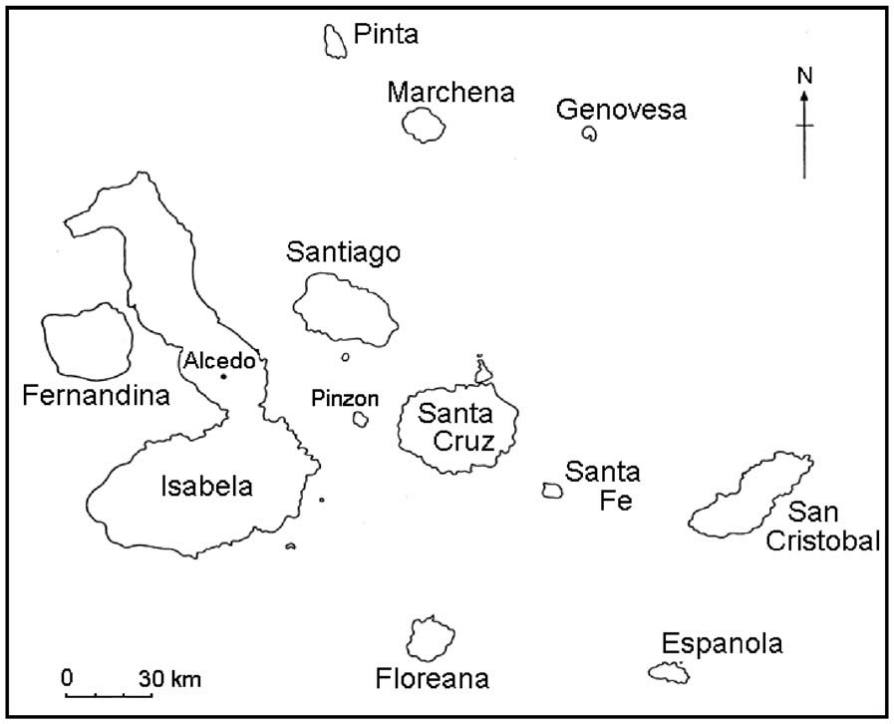
Map of Galapagos Islands. At the time of the collections used in this study, permanent inhabitants lived on San Cristobal and southern Isabela, and Floreana was occupied intermittently.

Our tissue sampling was restricted by CAS to specimens displaying at least two lesions, so as to not remove evidence of infection from these historically important specimens. In addition, we chose specimens from islands in a manner that maximized the number of samples from each of four islands for each focal taxon to evaluate geographic distribution. For finches, those islands were Floreana, Isabela, San Cristobal, and Santa Cruz, and for mockingbirds the islands sampled were Espanola, San Cristobal, Santa Cruz, and Santa Fe (Fig. 1). We excised samples from lesions from 59 specimens and placed them in sterile screw cap vials for transport. We replaced the cover of the working surface, scalpel blades, and gloves before examining each specimen to prevent cross contamination. Only Floreana, San Cristobal, and Isabela were inhabited by humans at the time of collection.

### Histopathology Studies

Each excised lesion was subdivided using aseptic technique and a representative portion of the lesion was processed for histopathology by ELB. The tissues were placed in 10% neutral buffered formalin for three days to re-hydrate and fix the tissue. Samples were then processed routinely, and stained with hematoxylin and eosin for light microscopic examination.

### Genetic Studies

PCR primers were designed by NKW for <150 bp segments of the virion core protease gene and the integral membrane protein gene that differed diagnostically for two previously characterized canarypox virus variants in Galapagos [12], two sequences of canarypox virus from Genbank, and fowlpox virus (details below). Direct sequencing on both DNA strands was performed on 17 amplicons and compared against a five-species reference alignment.

Genetic tests:

DNA was extracted from the remaining half of each excised lesion in a new lab that had never been used for DNA studies and was on a different floor from any other DNA labs. Poxvirus PCR amplicons from this study were stored in a separate building from the lab used for the CAS extractions and genotyping. We conducted extractions in a fume hood and cleaned all work surfaces with 5% bleach between extractions. We added 250 µL lysis buffer (0.1 M Tris-Hcl, pH 8.0, 0.1 M EDTA, 10 mM NaCl, 0.5% SDS) and used a sterile pipette tip to macerate the lesion, added 20 µL of Proteinase-K (final concentration, 1.0 mg/ml), and incubated at 65°C overnight (at least 6 h) before extraction with phenol/CHCl_3_/isoamyl alcohol (25∶24∶1). The final aqueous phase was dialyzed overnight against TNE_2_ (10 mM Tris, pH 7.4, 10 mM NaCl, 2 mM EDTA). A subset of samples was extracted by KP and HF in a different facility dedicated to ancient DNA using similar decontamination procedures. DNA was isolated using the same isolation buffer with 0.1 M DTT added, captured with glass micro-beads (QBiogene GeneClean Ancient DNA kits) following the manufacturer's protocol, then re-eluted in 50 µL H_2_O.

### Primer Design, PCR and Sequencing

We designed primers that would: (1) amplify regions <150 bp due to the likelihood that the CAS samples were degraded; and (2) discriminate between previously characterized virus sequences. We created an alignment in Clustal X [22] using the two 5,940 bp sequences from Gal1 and Gal2 variants present in contemporary Galapagos passerines (GenBank accession numbers AY631870 and AY631871), along with homologous sequences from two canarypox viruses (GenBank accession numbers D86731 and AY318871) and fowlpox virus (GenBank accession number AF198100) used in [12]. We used PrimaClade [23] to find low-degeneracy primer pairs <150 bp apart containing sites that varied between Gal1, Gal2 and the canarypox virus strains, the closest known relatives of Gal1 and Gal2 strains. Only two loci met these critera. The first was located within the virion core protease (CNPV111) gene (primer sequences: 1471F-ACYAGTATTCAGCAATTAATAGGACC and 1586R-AGGGCTGCAGATTTTTCGTAT; numbers correspond to 5′ location of the first base in the primer sequence in the five taxon alignment) and amplified a 115 bp fragment. Gal1 and Gal2 differed from the two canarypox strains at site 1535 (A in Gal1 and T in Gal2) and Gal1 and Gal2 differed at site 1563 (T in Gal1 and C in Gal2). The second locus was a 117 bp fragment located within the integral membrane protein (CNPV112) gene (primer sequences: 3521F-TGCTAGATCGTCGTTCGT and 3638R-CACTTTAGATTTCCTTATATATGCTG). Gal1 differs from Gal2 and the canarypox strains at site 3566 (A in Gal1 and G in the others) and Gal2 differs from Gal1 and the canarypox strains at site 3567 (A in Gal2 and G in the others). Gal1 and Gal2 differed from fowlpox at numerous sites at both loci.

We attempted to PCR-amplify each locus on the extracted CAS lesions and three positive controls (from a Darwin's finch, a yellow warbler, and a Galapagos mockingbird) from [12]. Each tube contained 32.5 µL sterile deionized H_2_O, 3 µL of each primer (10 µM), 1.5 µL of 25 mM MgCl_2_, 5 µL of 10× *Taq* Polymerase Buffer (Sigma), 1 µL Bovine Serum Albumin, 1 µL 10 mM dNTPs, 1 µL of *Taq* polymerase (Sigma) and 2 µL of template DNA. Alternatively, an antibody-bound *Taq* buffer system (Amplitaq gold, Applied Biosystems) was used in a 25 µL final volume reaction. The PCR cocktail and template DNA from the CAS samples were added to the PCR tubes in a room that was not used for poxvirus sample or amplicon storage or for poxvirus PCR-amplification. These closed tubes (including a negative control) were then transported on ice to the Parker Lab where template DNA for the positive controls was added to those tubes and the samples were placed in the thermocycler. An annealing temperature of 48°C was used for the first primer pair (1471F and 1586R) and 53°C for the second (3521F and 3638R). Each program ran for 35 cycles under standard reaction conditions with a final 7 minute extension at 72°C. Amplicons were verified on 1–2% TBE agarose gels stained with ethidium bromide and visualized under UV light. If bands of the expected size appeared, we purified those products using a QIAQuick PCR purification kit. All PCRs were rerun at least twice and as many as seven times for samples yielding ambiguous results. To be scored positive, a sample must have amplified at least twice. Ultimately, all samples were either consistently negative or amplified at least twice, in both the UMSL laboratory and the ancient DNA facility.

Direct sequencing was performed on both strands of 17 amplicons using the same primers with ABI PRISM® BigDye Terminator PCR cycling conditions and sequenced on an Applied Biosystems 3100 DNA Analyzer (Applied Biosystems Division, Foster City, CA). Raw sequence chromatograms of forward and reverse strands were assembled in Seqman II (DNASTAR, Inc., Madison, WI, USA). The entire length of each strand was evaluated by eye. Poor quality data and primer sequences were trimmed from both strands. Eleven samples yielded good sequence data at the four diagnostic variable sites in the virion core protease and integral membrane protein genes. Comparing against the five-taxon reference alignment, these positive individuals were identified as being infected with Gal1 or Gal2 variants (GenBank accession numbers AY631870 and AY631871).

## Results

### Apparent Prevalence of *Avipoxvirus*

Of the 3607 CAS specimens examined, 226 (6.3%) displayed gross cutaneous lesions consistent with *Avipoxvirus*, showing raised nodules of smooth or ulcerated surface with well-defined margins, from 1 to 4 mm in diameter. Lesion prevalence by island ranged from 0.011 to 0.18 (Table 1). The highest apparent prevalences on islands where more than 100 birds were sampled were on San Cristobal (80 of 554 or 14.4%) and Santa Fe (22 of 198 or 11.1%); these values are biased by samples from mockingbirds, which displayed lesions on 34 of 137 (24.8%) specimens from San Cristobal and 19 of 72 (26.4%) from Santa Fe. Mockingbirds had the highest apparent prevalence: 124 of 704 (17.6%) displayed lesions on 13/15 (86.7%) islands. Of the 266 specimens examined in ZSM, none displayed any lesions consistent with poxvirus infection.

**Table 1:** Passerine birds examined in the California Academy of Sciences collection of Galapagos birds, 1898–1906.

	Finches		Mockingbirds		Total	
ISLAND	Examined	With Lesions	Examined	With Lesions	Total Examined	With Lesions (%)
Daphne	23	1	1	0	24	1 (0.042)
Darwin	37	0	8	2	45	2 (0.044)
Espanola	206	7	75	15	281	22 (0.078)
Fernandina	46	1	16	2	62	3 (0.048)
Floreana	441	7	42	3	483	10 (0.021)
Genovesa	82	3	19	3	101	6 (0.059)
Isabela	567	11	90	9	657	20 (0.030)
Marchena	81	0	36	4	117	4 (0.034)
Pinta	115	2	31	6	146	8 (0.055)
Pinzon	69	1	0	0	69	1 (0.014)
Rabida	19	0	31	9	50	9 (0.18)
Santa Cruz	353	9	120	15	473	24 (0.051)
S Cristobal	417	46	137	34	554	80 (0.144)
Santa Fe	126	3	72	19	198	22 (0.111)
Santiago	238	10	18	3	256	13 (0.051)
Wolf	83	1	8	0	91	1(0.011)
TOTAL	2903	102	704	124	3607	226 (0.063)

The finches include species in *Geospiza*, *Camarhynchus*, and *Platyspiza* genera, and mockingbirds include all four *Mimus* species in Galapagos. Prevalence is the proportion of specimens displaying lesions that could have been caused by the *Avipoxvirus,* prior to the testing of a subset of these specimens.

The 59 tissue samples taken from finch (from 4 islands) and mockingbird (from 4 islands) specimens represented six major islands overall, three of which were inhabited at time of collection (San Cristobal, Isabela, Floreana) and three of which were not (Espanola, Santa Cruz, Santa Fe: Table 2).

**Table 2:** Test results from histopathology and PCR for lesions excised from 59 specimens in the California Academy of Sciences 1898–1906 collection from Galapagos.

	Finches		Mockingbirds		Total	
ISLAND	Tested	Positive	Tested	Positive	Tested	Positive (%)
Espanola			9	0	9	0 (0)
Floreana	3	0			3	0 (0)
Isabela	3	3			3	3 (1.0)
Santa Cruz	2	0	6	0	8	0 (0)
S Cristobal	17	12	12	5	29	17 (0.59)
Santa Fe			7	1	7	1 (0.14)
Total	25	15	34	6	59	21 (0.36)

Histopathology and PCR agreed on 16 of 21 positives, two PCR positives lacked sufficient material for histopathology, and three samples positive by histology did not amplify by PCR and were counted as positive (see text).

### Histology & Genotyping

**Figure 2:**
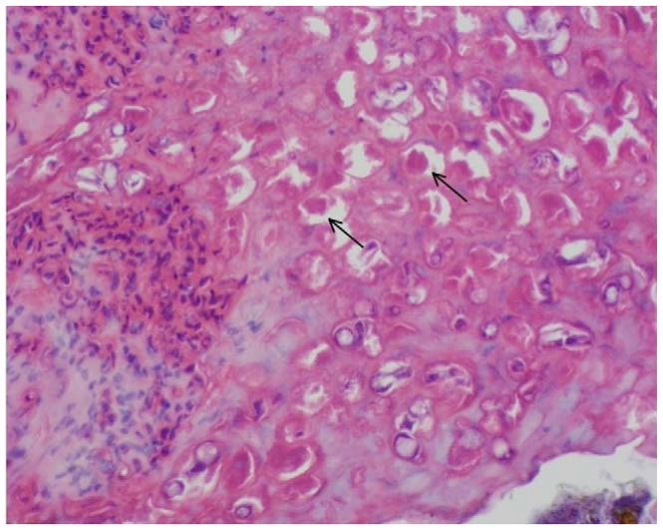
Histopathology of lesion from Medium Ground Finch (*Geospiza fortis*). This specimen from the California Academy of Science was collected on San Cristobal Island in Galapagos in 1905. Inclusion bodies diagnostic of avipoxvirus occur throughout; two are indicated by arrows.

Despite the age and condition of the samples, histologically pathognomonic pox lesions were diagnosable. Pox viral infection was diagnosed independently of the PCR-based diagnoses in 19 of the 59 specimens by hyperplastic epidermis and marked ballooning of the keratinocytes. The majority of the keratinocytes contained large eosinophilic intracytoplasmic inclusion bodies that distorted or displaced the nucleus (Bollinger bodies: Fig. 2). Diagnoses for pox-negative birds (n = 27) included feather follicle cysts, non-specific dermatitis, and post mortem fungal colonization. Thirteen samples contained insufficient tissue for accurate analysis by histology.

Eighteen of the 59 specimens we sampled were positive by PCR, while 41 did not amplify and were scored PCR-negative. Sixteen of the PCR-positive samples were also positive by histopathology, and the other two PCR-positive samples did not have sufficient material for histopathology. None of the PCR-positive specimens were negative by histopathology. Conversely, 16 of the histo-positive specimens were also positive by PCR, while 3 histo-positive specimens were not positive by PCR. We scored these three apparent discrepancies as positive. This also indicates a very low contamination rate from more recently collected specimens or amplicons, given the nearly complete overlap between histopathology and PCR based tests and the fact that no PCR-positive specimen was judged histo-negative. Overall, 21 (35.6%) of the 59 specimens were scored as positive (Table 2).

For the 34 mockingbirds sampled, positives were nonrandomly distributed across islands (p = 0.05, Fisher's Exact Test): five from San Cristobal (of 12 sampled, or 42%) and one from Santa Fe (of 7, 14.3%) were positive (Table 2), while 15 from Espanola and Santa Cruz were negative. For the 25 finches sampled, 12 of 17 (70.6%) from San Cristobal and all three from Isabela were positive while five from Santa Cruz and Floreana were negative (p = 0.005 Fisher's Exact Test). Summing across taxa, finch and mockingbird specimens on islands inhabited by humans at the time of collection (Floreana, Isabela, San Cristobal) were significantly more likely to be positive for the avipoxvirus (20 of 35, 57%) than those on islands not inhabited by humans (1 of 24, 4.2%) (p<0.001, Fisher's Exact Test). Six of the positive finches on San Cristobal had *Avipoxvirus* DNA sequence identical to Gal2 [12] at the diagnostic virion core protease gene and the integral membrane protein gene; a seventh positive finch was identical to Gal1 [12] at both genes. Of four pox-positive mockingbirds successfully sequenced, two were identical to Gal1 and two were identical to Gal2. These four diagnostic sites in two genes of course do not preclude the possibility of other strains present historically or currently, and our ongoing work will further describe variability in historic and extant strains.

Had avipoxvirus been present in 1891 and 1897 at the apparent prevalences detected in 1898–1906 (Table 1), the probability of detecting no birds with lesions is ≪0.0001.

## Discussion

These results indicate that 110 years ago, 64 years after the *Beagle* anchored and Charles Darwin collected specimens in the Galapagos Islands, *Avipoxvirus* was present in its endemic birds. At that time the virus was heavily concentrated on the human-inhabited islands, particularly San Cristóbal, which was the most heavily human-populated island at that time, compared to the much smaller settlements on Isabela and Floreana [18]. This is consistent with field notes of the 1905–1906 collectors, whose journals made reference to frequent “diseases of the feet” in birds on San Cristobal [24]. The other positive samples were from Isabela (3 specimens), which was also inhabited at that time, and from Santa Fe (1 specimen), which was not. That none of the ZSM specimens collected in 1891 and 1897 displayed lesions suggests arrival of the disease in Galapagos shortly before the 1898–1899 sampling expedition.

The mode by which the *Avipoxvirus* infecting passerine birds in Galapagos initially arrived is unknown. There is no evidence that these infections derived from the poxvirus in domestic farmyard birds; in both Galapagos and Hawaii, the virus infecting the domestic chickens is distinct from that infecting passerines, and the form in passerines clusters with previously described canarypox virus present elsewhere [11], [12]. It is possible that early settlers brought pet caged birds and so introduced the virus. It is also possible that the initial arrival was a natural event with an infected migrant passerine such as the Bobolink (*Dolichonyx oryzivorus*), the only passerine regularly seen (primarily on San Cristobal) during its annual migrations between North and South America. We regard arrival with an insect vector as unlikely, partly because the principal mechanical vectors were first recorded relatively recently (*Culex quinquefasciatus* in 1985, *Aedes aegypti* in 2001, *Simulium bipunctatum* in 1989 [25]). Regardless of the mode of arrival, we expect that further research using other collections will support an arrival date shortly prior to 1899 (our earliest positive specimen) on San Cristóbal, and propose that its presence on Isabela in 1905–1906 was associated with human traffic between colonies that would accelerate the rate at which inadvertently transported virions accumulated in particular locations, resulting in localized outbreaks and sustained presence of the virus. In addition, the behavior of birds changes in the presence of human settlements, as they aggregate at sources of food and water, accelerating disease transmission [26]. The regular visits of whalers, fishermen and buccaneers among islands for hundreds of years was perhaps less likely to result in a sustained local epidemic due to its more haphazard localization and lengthy times at sea between landings, during which virions would be rinsed from footwear and decks. The virus likely spread naturally among islands as well, given movement of finches among islands [27] and even more frequent movement of other susceptible species such as the endemic Galapagos dove [28], and we suggest that the single positive sample on Santa Fe in 1906 was early evidence of this natural movement.

The other possible mode of movement of the virus is with the arthropod vectors that may mechanically transport virions from one blood meal to the next. The arrival of the bird-biting *Culex quinquefasciatus* in Galapagos implicates this species in viral transmission between birds. However, this mosquito is not thought to wander widely, and its distribution in Galapagos is currently restricted to sites having fresh water [29]. The other more widespread black saltmarsh mosquito (*Aedes taeniorhynchus*) is known to take at least occasional blood meals from birds, but its strong population differentiation among islands and habitats suggests very short dispersal distances [30]. For these reasons we think it unlikely that arthropod vectors have played an important role in moving this virus among islands.

Today, repeated localized travel occurs regularly among inhabited and uninhabited islands in the form of tourism, Park management visits, scientific research, and permitted and illicit hunting and fishing. And since most of these diverse groups use common landing sites on both inhabited and uninhabited islands, they have likely contributed to the presence of this virus and other pathogens across the archipelago. Of these groups, only scientists undergo rigorous quarantine procedures to minimize or eliminate transport of organisms between islands. In modern times, pox-like symptoms are reported regularly in birds on Santa Cruz, Isabela, San Cristobal, and Floreana [12], [15], [17], and in much lower prevalences on uninhabited islands of Santiago and Marchena (Jimenez-Uzcategui, pers. comm.) and during extreme weather events such as El Nino on the uninhabited island of Genovesa [14].

Terrestrial indigenous Galapagos birds today number 28–30 taxa from an estimated 14 successful colonization events; radiations have followed only in the mockingbirds (4 species) and finches (at least 13 species). Their early colonizations and subsequent diversifications [31], [32] suggest that they have existed in isolated or semi-isolated subpopulations, features that make them attractive subjects for studies of evolutionary mechanisms. However, isolation in small insular populations may also leave them more vulnerable to any pathogens that should arrive [33] due to loss of genetic variability in small populations and loss of co-evolved immune responses. In addition, factors associated with disease-induced extinction (small population size, availability of reservoir hosts, and ability of the pathogen to survive outside of a host [34]) are relevant to many Galapagos bird populations in their relationship with *Avipoxvirus*. The critically small populations of the Floreana Mockingbird (*Mimus trifasciatus*: 85–225 birds; [35]) are strikingly depauperate in genetic variability [36]. Genetic drift acting on all isolated mockingbird populations in the Galapagos [37] and the known susceptibility of the Galapagos Mimidae to *Avipoxvirus* [14], [15] may increase extinction risk should conditions favor an outbreak, such as during the next ENSO event, when extensive rains cause irruptions of arthropod vectors of *Avipoxvirus*.

**Figure 3:**
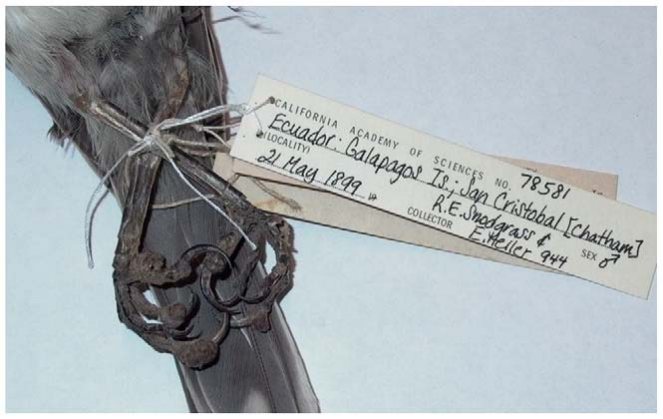
Chatham Mockingbird (*Mimus melanotus*) collected in May 1899 from San Cristobal Island (in CAS collection). The lesion on the center left toe was sampled, and was positive for *Avipoxvirus* by histopathology and PCR.

**Figure 4:**
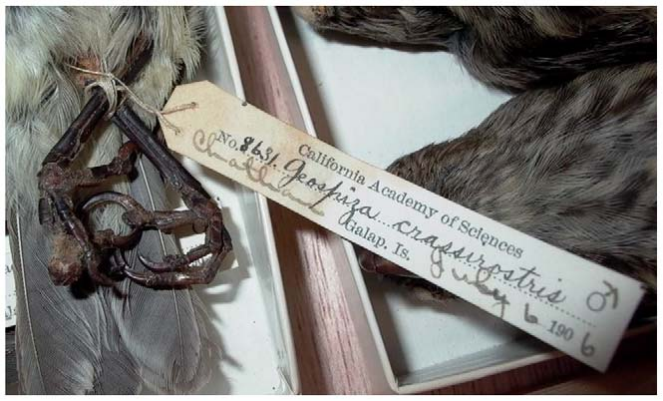
Vegetarian Finch (*Geospiza crassirostris*) collected in July 1906 from San Cristobal Island (in CAS collection). The lesion on the center left toe was sampled, and was negative for *Avipoxvirus* by histopathology and PCR.

These results also indicate that diagnosis of *Avipoxvirus* should be made carefully, and that visual inspection for cutaneous lesions (*e.g*., [17], [38]) is not sufficient, as fewer than half of the lesions we tested were positive for *Avipoxvirus*. It could be argued that true pox infections may be missed by both PCR and histopathology in such historic samples, but several were given clear alternative diagnoses in histopathology; in other words, causative agents other than *Avipoxvirus* were provided for the symptoms. At the very least, these preliminary diagnoses should be suggested as “pox-like” (e.g., [15]), but in no case should further analyses be based upon the presumption of true pox infections without confirmation. We provide images of the very similar appearance of lesions on a mockingbird from San Cristobal in 1899 (Fig. 3) that was positive by both histopathology and PCR, and a vegetarian finch from San Cristobal in 1906 (Fig. 4) negative by both tests.

Regretfully, the Galapagos Islands are now inhabited by the same three elements that triggered a massive decline of endemic birds in Hawaii: the *Avipoxvirus*; competent vectors in *Culex quinquefasciatus* and perhaps other mosquitoes; and the recently detected *Plasmodium* blood parasite [39]. We show here that *Avipoxvirus* has been on the islands at least since 1899. *Culex quinquefasciatus* was first documented in the 1980′s and later confirmed to have breeding populations [40], now residing near areas of human settlements and fresh water on several islands [29]. With the detection of *Plasmodium* in Galapagos penguins, we are working hard to understand the transmission dynamics among endemic bird populations for these two pathogens and their associated vector communities. We remain optimistic that the Galapagos avifauna can avoid the declines and extinctions suffered in Hawaii, by effective management practices that require a more thorough understanding of the roles played by each of the three elements, their individual histories and dynamics, and their interactions.
